# Correlation between Rheological Fatigue Tests on Bitumen and Various Cracking Tests on Asphalt Mixtures

**DOI:** 10.3390/ma14247839

**Published:** 2021-12-17

**Authors:** Muhammad Aakif Ishaq, Filippo Giustozzi

**Affiliations:** Civil and Infrastructure Engineering, School of Engineering, RMIT University, Melbourne, VIC 3001, Australia; s3647650@student.rmit.edu.au

**Keywords:** fatigue, cracking, bitumen, asphalt, tensile strength, fracture energy, dissipated energy

## Abstract

Accurate characterisation and appropriate binder selection are essential to increase the load-induced cracking resistance of asphalt mixtures at an intermediate temperature. Hence, the primary goal of this study was to correlate the cracking resistance exerted by the binder with the cracking performance of asphalt mixtures. The laboratory-based experimental plan covered various types of laboratory tests specified by various agencies and road authorities to study the correlation of a neat bitumen and five polymer-modified binders with their corresponding asphalt mixtures. The fatigue life of the binders was assessed through a Linear Amplitude Sweep (LAS) test and statistically correlated with various load-induced cracking parameters from the indirect tensile test, semi-circular bending (SCB) test, and four points bending beam test (FPBB) of asphalt mixtures at 25 °C. Binders and mixes were further grouped depending on their polymeric family (i.e., modified with a particular type of polymer) to validate their statistical correlation. The indicator that mostly correlated the binder properties with the asphalt mixture properties is the secant modulus from the SCB test. Fatigue parameters obtained through LAS better explain the asphalt fatigue performance obtained through FPBB; specifically, asphalt tests at high strain levels (e.g., 400 micro strain) better correlate to the LAS fatigue parameter (Nf).

## 1. Introduction

Asphalt pavements are affected by three major deterioration mechanisms: moisture damage, cracking, and permanent deformation [1,2,3,4]. Traffic-induced cracking of flexible pavements manifests as alligator cracking on the pavement’s surface due to recurrent stresses and strains produced by cyclic loading at an intermediate temperature [5,6,7]. Cracking on pavements depends on the road pavement structure (i.e., layer thickness, stiffness modulus, and rheological properties of bitumen), traffic, environmental conditions, and the time-dependent variation (aging) of bitumen [8]. In addition to the deterioration of the pavement’s structural integrity, cracking also reduces the road functionality, including safety, comfort, and operating expenses for the user [9].

Although many variables influence the cracking behaviour of asphalt mixes (i.e., ambient conditions, mixture properties, traffic loading, binder, and aggregate properties), the binder is said to perform the most critical function [6,10,11]. The vulnerability to cracking increases when the bitumen stiffens due to environmental aging, increasing its capacity to form cracks from repeated traffic stresses [12]. The durability of asphalt pavements can be enhanced by appropriate binder selection and precise characterisation of the asphalt binders [13,14].

Bitumen modification, an effective tool for making asphalt roads more durable and less susceptible to rutting, has advanced in recent decades [15,16]. However, finding a binder that can endure cracking remains a problem due to a lack of research investigating how polymer-modified binders resist long-term fatigue cracking when incorporated into asphalt mixes in the field under actual traffic loading. One explanation for this may be associated to a deficiency in testing methods that allows for assessing binder’s cracking characteristics. Most of the past advancements in rheological testing are limited to the domain of linear viscoelastic behaviour with relatively small deformations [17].

Moreover, several agencies worldwide continue to use empirical tests to characterise binder performance at intermediate temperatures. This situation has been exacerbated further by the recent invention of several new asphalt binders, including hybrid (i.e., combining more than one polymer) binders, binders that are specifically modified using waste materials (crumb rubber, plastic, etc.), and high-performance binders for specialised applications (for example, airport pavements) [18,19,20,21,22]. Conventional and commonly used binder tests that may be generally associated with cracking resistance comprise of i) traditional empirical tests, such as direct tensile test, ductility, and penetration; ii) time sweep tests; iii) linear viscoelastic rheology using a dynamic shear rheometer (DSR) and more advanced rheological tests, such as a LAS test [23,24].

In the Superpave study, the G* sinδ parameter (phase angle δ and complex shear modulus |G*|) is used to quantify the fatigue resistance of the asphalt binder [25]. For strain-controlled testing, Strategic Highway Research Program (SHRP) researchers assumed that a lower dissipated energy of each loading cycle [w_i_ = πεG* sinδ] correlates to a lesser accumulation of distress, hence implying that asphalt with a lower G* sinδ value would be more resistant to fatigue cracking. The use of G* sinδ to evaluate fatigue cracking performance of polymer-modified asphalts has been associated with some drawbacks by several studies [26,27,28,29,30]. The complex polymer structures are not activated when such material characteristics are measured predominantly in the small strains domain and inside the linear viscoelastic zone; therefore, the full advantages of polymer modification are not highlighted by this parameter [27]. However, in a limited number of investigations, the G* sinδ parameter strongly correlated with the fatigue cracking of polymer-modified asphalt mixtures tested using various testing techniques [15,25,31,32].

The linear amplitude sweep (LAS) is considered a more advanced test in the fatigue characterisation of bitumen. It was devised as an expedited fatigue test to substitute for the time-sweep test [33]. The strain amplitude is gradually increased in a systematically linear method to achieve faster damage of the sample. The LAS test analysis is based on the viscoelastic continuum damage (VECD) model, which has been widely used to characterise and predict the fatigue performance of asphalt mixes [34,35,36,37]. The subsequent damage characteristic relationship can be utilised in the strain-based fatigue simulation, which enables fatigue life to be predicted at any given stress amplitude, frequency, and temperature. The results of fatigue prediction were shown to have a reasonable correlation with field cracking measured in asphalt pavements [38]. Table 1, Table 2, Table 3, Table 4 and Table 5 summarise the critical literature on correlations between various binder properties and cracking of asphalt mixes. 

Similar to binders, various tests are available to evaluate the cracking of asphalt mixes, which assist in predicting the performance of the pavement in the field. Cracking at intermediate temperatures can be evaluated using crack initiation (fatigue cracking) and the crack propagation concept (fracture resistance properties). The need to study these two mechanisms is because of the elastic recovery properties of binders imparted by their viscoelastic nature. Hence, a crack (fracture) produced by a single traffic-induced load does not lead to a significant cracking; instead, recurrent stresses and strains by traffic-induced loads result in significant cracking that manifests itself as alligator cracking (fatigue cracking). Therefore, knowing the potential of crack initiation and then crack propagation can help characterise the asphalt mixtures more comprehensively. 

Many laboratory testing protocols have been developed to quantify cracking resistance of asphalt mixtures whether under dynamic or static loads, but no unique consensus has been achieved on which one should be universally adopted [39]. Among the many tests are the Fenix test [40], single-edge notched beam [41], semi-circular bending test [42,43,44], dog-bone direct tension [45], overlay test [46,47], indirect tension test [48,49,50], disc-shaped compact tension [49], and IDT asphalt cracking test [51]. For dynamic testing specifically, a more detailed explanation around the benefits and drawbacks of fatigue testing methodologies can be found in [39,52]. 

IDT and SCB are some of the candidate tests for studying the crack propagation phenomenon by characterising the fracture resistance properties of the asphalt mixtures [53,54,55,56,57]. The IDT and SCB test techniques are typically employed to determine an asphalt sample’s maximum tensile strength, slope, and fracture energy at intermediate temperatures. SCB test method is based on the fracture mechanics concept with low variability in test results and has been used to determine the fracture resistance of the asphalt mixtures. The IDT test technique is utilised as a performance-based quality control tool during the design and manufacture of asphalt mixtures and for estimating the cracking potential [58,59]. Both IDT and SCB tests are elementary and can be carried out using standard laboratory equipment, frequently available in any asphalt plants’ laboratory.

The most popular tests for investigating the fatigue cracking potential are categorised as (i) simple flexure tests (designed to create a direct connection between fatigue life and stress/strain by subjecting beams to pulsing or sinusoidal loads in either a third or centre-point layout, rotating cantilever beams, and trapezoidal cantilever beams), such as rotating cantilever, two-point loading on trapezoidal specimens, three-point loading, and four point loading on prismatic specimens; (ii) direct axial loading tests (pulsing or sinusoidal loads applied uniaxially with or without stress reversal), such as push–pull or tension–compression on cylindrical specimens; and (iii) diametral loading test (pulsing loads to diametral cylindrical specimens), namely, uniaxial repeated loading tests on cylindrical specimens. These tests use pulsing loads on cylindrical specimens in the diametral direction to build a direct link between fatigue life and stress/strain [17,60,61,62,63,64]. Generally, factors that differentiate among suitable fatigue cracking laboratory tests are convenience or user-friendliness, sample manufacturing time, test duration, cost efficiency, correlation with field performance, multi-data generation capabilities, and adaptability to well-established asphalt mix design and screening procedures.

The VECD approach is gaining popularity as a viable tool for determining the durability of asphalt mixtures against fatigue cracking during uniaxial tests (i.e. push–pull tests). The applicability of constitutive models, such as VECD, is favourable over the lengthier FPBB. In addition, uniaxial fatigue tests promote a constant stress state in the specimen section, hence better representing asphalt’s fundamental properties. As a downside, uniaxial tests commonly require parallel surfaces of the samples to run, hence requiring cutting of the sample and consequent gluing to the testing jig. Seitllari and Kutay have recently devised a novel technique for determining the fatigue cracking performances of asphalt mixtures, termed as the three-point bending cylinder (3PBC) test [57]. The novel 3PBC testing configuration adopts the VECD method but does not require any cutting, gluing, or creating a notch in the samples. In addition, it offers some interesting features for pavement design, such as the estimation of Poisson’s ration from the test data. Despite the ongoing efforts, many of these techniques are still under investigation, mostly to assess their ability in capturing changes in mix design parameters and variation in results.

The flexural beam fatigue test, suggested by SHRP A-003A, is presently used by several agencies worldwide as a performance test for the asphalt mixtures’ fatigue cracking assessment at an intermediate temperature [65,66,67,68]. Both constant stress and constant displacement modes can be used to perform FPBB. The constant displacement mode, often known as the constant strain mode, has become the standard form of testing over time. Unlike previous tests, the four point bending test (even referred to as the third point flexure test) was intended to focus on the specimen failure in a domain of uniform bending moment [69]. The advantage of third-point loading over centre-point loading is that the bending moment is constant through the middle third of the specimen; therefore, any weak region owing to non-uniform material qualities will be shown in the test results [70]. FPBB can interpret results using fracture mechanics and dissipated energy techniques to explicitly study the cracking initiation under repeated traffic load.

A comprehensive literature review was conducted to cover the correlation between bitumen and asphalt mixtures, testing parameters in the form of the Pearson correlation coefficient (R^2^, the proportion of the variation in the dependent variable that is predictable from the independent variable) and considering various tests at different conditions (Table 1, Table 2, Table 3, Table 4 and Table 5). Information on the testing and material types has also been reported in these tables. 

In Table 1, the binder and its fraction percentage were correlated with various parameters of asphalt mixtures. The correlation of the binder content (%) and asphaltenes (%) from a Saturate, Aromatic, Resin and Asphaltene (SARA) analysis with the various asphalt mixtures’ test parameters found in the literature was weak to moderate. Similarly, Table 2 shows that various miscellaneous tests (not necessarily related to cracking) conducted on binders were correlated with cracking parameters of asphalt mixtures. The strength of the correlation was variable (weak, moderate, and strong) depending on the particular study considered.

In Table 3, rheological parameters from the time sweep test on binders were correlated with various cracking parameters of asphalt mixtures tests. The strength of the correlation was found as moderate to strong, whereas only a few studies reported a weak to moderate correlation.

In Table 4, the SHRP fatigue cracking parameter from frequency sweep tests on binders was correlated with various cracking parameters from asphalt mixture tests. The correlation was found moderate to strong except in one study where it was found weak.

Table 5 shows the correlation between parameters from the LAS test on binders with various cracking parameters of asphalt mixture tests. Correlation was found moderate to very strong except on a few studies where it was weak to moderate.

The previous Table 1, Table 2, Table 3, Table 4 and Table 5 show that bitumen tests used to determine fatigue properties and asphalt mixture tests have varying degrees of correlation. Conventional bitumen tests have limited significance in predicting the asphalt mixture’s fatigue properties. The SHRP fatigue rheological parameter is only limited to the analysis of linear viscoelastic behaviour under small deformations—one of the primary justifications for the poor correlation, especially for polymer-modified bitumen (PMB). The time-sweep test demonstrated a significant correlation with the fatigue characteristics of asphalt mixes. However, given the uncertainty in the testing period and reduced testing repeatability, the time sweep test is not a feasible approach for specifying asphalt binder fatigue resistance [38]. 

Although only a few studies showed a significant correlation between asphalt and binder tests, others found little to none; this was primarily dependent on bitumen content, polymer types used in modification, aggregate gradation, testing technique, and loading and environmental conditions utilised in the research studies. The usage of emerging polymer-modified bituminous binders (highly modified, plastomer-modified, hybrid-modified, modified with recycled material) may contribute to this variability; therefore, additional research is needed to correlate bitumen and asphalt intermediate temperature performance. There is currently limited data or correlations comparing binders and asphalt mixes made from plastomers, elastomers, polymeric compounds with fibres, and amino-wax-based additives. Furthermore, the existing literature shows no direct association between binder fatigue life determined by the LAS method and asphalt cracking determined through IDT, SCB, and FPBB tests. The present study evaluates a variety of polymer-modified binders and their performance in asphalt mixes.

The main goal of this study is to determine the statistical correlation between fatigue cracking parameters of different asphalt binders as measured by the LAS parameters and the cracking resistance of asphalt mixes produced with the same binders and tested using IDT, SCB, and FPBB techniques. Various polymer-modified binders were tested under varied testing conditions to determine the correlation between bitumen and asphalt tests (i.e., temperature and stress level).

## 2. Materials and Methods

This research used one neat bitumen and five different polymer-modified binders to examine the correlation between the binders and their respective asphalt mixtures. These polymers are comprised of plastomers (6% by weight of the binder), elastomers (approximately 6% by weight of the binder), polymeric compounds of elastomer and micro-cellulose fibre (0.3% by weight of the aggregate), polymeric compounds of plastomers/elastomers and micro-cellulose fibre (0.3% by weight of the aggregate), and amino-wax based additives (0.3% by weight of the binder) used in bitumen modification that reduces mixing and the compaction temperature of asphalt mixtures. Table 6 outlines the material characteristics and the methodology used for blending various polymer binders and corresponding mixtures in this research study. The dosages of various polymers/additives and their mixing and compaction conditions were selected based on the manufacturer’s recommendations and previous outcomes from literature studies on similar types of products and mixes. It should be noted that this research aimed to study the correlation between various tests looking at the cracking properties of bitumen and asphalt mixtures for a wide range of blends rather than to assess their relative performance. Hence, the different polymer content among the various mixes does not affect the main scope of the analysis but rather enhances the variability among samples to possibly ascertain greater statistical significance when looking at the correlation between tests.

Cylindrical IDT and SCB samples and prismatic beam-like samples were compacted to achieve 5 ± 0.5% air voids. Asphalt mixtures were manufactured at 5% bitumen content (by weight of the mix).

**Figure 1:**
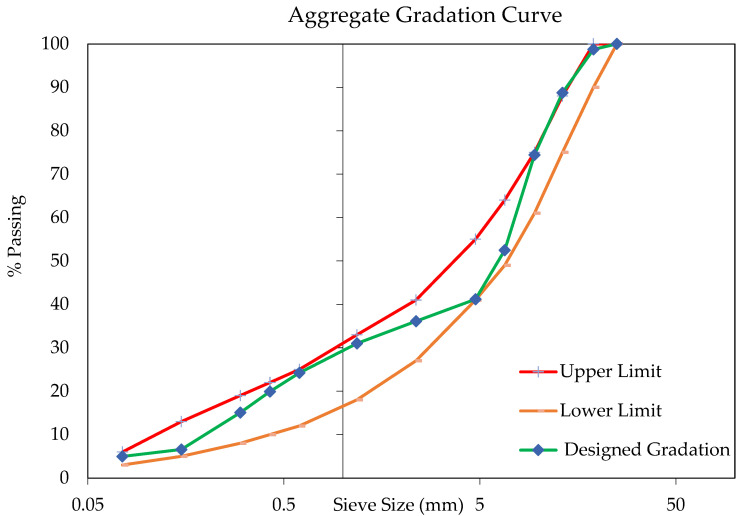
Combined aggregate gradation curve of the asphalt mixtures.

The mixed aggregate gradation is shown in Figure 1 and reflects the proportion identified by Australian standards [86] for an intermediate asphalt layer.

## 3. Experimental Plan

**Figure 2:**
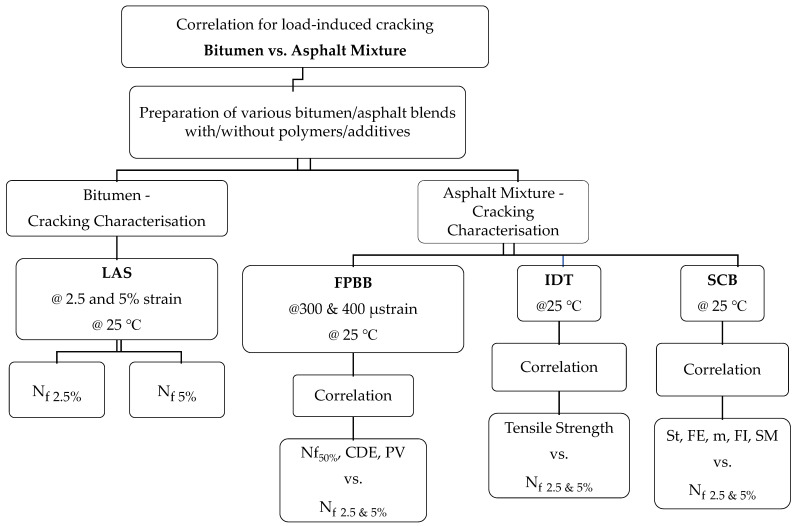
Experimental plan.

A total of four different types of testing, one on the binder and three on the asphalt mixtures, were conducted in this research. The methodology followed to develop the correlation among the cracking properties of the various binders, and their corresponding asphalt mixtures is given in Figure 2. The research was further divided into two major parts. Phase 1 investigated the fatigue performance of the binders via the LAS test (AASHTO T 391), which considers a 35% reduction in the initial modulus. The number of cycles to failure was evaluated at two different strain levels (2.5% and 5%) in the LAS test. Phase 2 assessed the cracking initiation and propagation potential of asphalt mixtures produced using the LAS test’s respective binders. Three different types of tests, namely, IDT, SCB, and FPBB, were carried out. 

Apart from the interest in determining how bituminous binders’ cracking properties may aid in the accurate prediction of the cracking potential of asphalt mixes at an intermediate temperature, this study was unique in that it investigated how different types of blends (plastomers, elastomers, polymeric compounds with fibres, amino-wax based additives, and unmodified bitumen) expressed the bitumen–asphalt correlation under varied testing conditions (i.e., loading rate and loading modes). Blends were further grouped based on blend composition and diversity of materials to investigate how this affected the correlation; for example, the “PMB family” includes all blends made up from various polymers (plastomers, elastomers, and polymeric compounds with microfibres). The most comprehensive group was “All-Sources” that includes all modified blends (four polymer-modified blends + one modified blend with an additive) and a blend without modification. 

### 3.1. Linear Amplitude Sweep (LAS) Test

The LAS test comprises an initial frequency sweep test followed by the amplitude sweep test [38,82]. A frequency sweep test with a strain amplitude of 0.1% and frequency range from 0.1 Hz to 30 Hz was performed according to AASHTO T 391 to obtain the undamaged material characteristics and fatigue law parameter (α) [87]. The second part of the test was then carried out in oscillatory shear mode at a constant frequency of 10 Hz and 25 °C. Strain was increased linearly from 0.1% to 30% throughout 3100 cycles of loading, for a total test time of 310 s. Three replicates were tested at each testing condition. The viscoelastic continuum damage (VECD) mechanics model [88] was used to compute the A and B parameters of the fatigue law (Equation (1)). In the LAS test, failure criteria are defined as a 35% decrease of the initial modulus of the binder.

(1) $$\;\;\;\;\;\;\;\;\;N_{f}=A_{35}{\left(\gamma_{max\;}\right)}^{-B}\;\;\;$$

where:

A and *B*: Parameters of the VECD model associated with the material property
*N_f_*: Fatigue failure life
*γ_max_*: Maximum shear strain for the given pavement structure

### 3.2. Indirect Tensile (IDT) Test

A cylindrical specimen was constantly compressed at 50 millimetres per minute during an indirect tensile test until it failed. A gyratory compactor was used to fabricate specimens with a diameter of 100 mm and a thickness of 65 mm, with air voids of 5 ± 0.5%. The test was conducted at 25 °C according to ASTM D6931-17, and three replicates for each asphalt mix were tested. The failure strength (*ITS*) was calculated using Equation (2).

(2) $$ITS\;\left(St\right)=\frac{2000P}{\pi Dt}.\;$$

where *ITS* = Indirect Tensile Strength (kPa), *P* = maximum load (N), *t* = thickness of specimen (mm), and *D* = diameter of specimen (mm).

### 3.3. Semi-Circular Bending (SCB) Test

The Illinois Flexibility Index Test (I-FIT procedure), also referred to as Illinois SCB (IL-SCB), was performed at an intermediate temperature of 25 °C according to AASHTO TP-124 [89]. A three-point bending loading setup was used to apply tensile stresses to a half-cylindrical test specimen (150 mm × 50 mm in diameter and thickness, compacted at air voids of 5 ± 0.5%) with a notch in the centre. The sample was loaded with a 50 mm/min constant deformation rate until failure [43,90,91]. The flexibility index (FI), fracture energy (FE), fracture strength (St), slope (m), and secant modulus (s.m) were the primary outcomes of the test method. Three samples for each material were tested under similar environmental and loading conditions.

#### Data Analysis Technique

**Figure 3:**
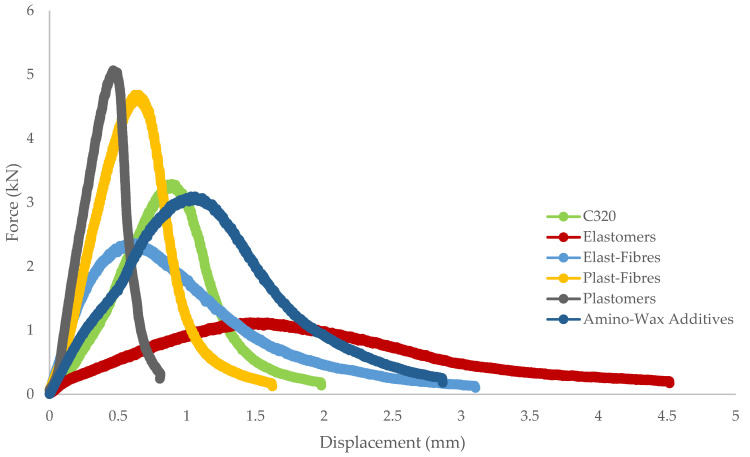
Typical load–displacement curves for various mixes.

The load–displacement curves for the six studied mixes are shown in Figure 3. It is interesting to observe that various materials exhibited different behaviours before and after the failure of the specimen. Less stiff and more elastic mixtures (elastomers, amino wax-additives) exhibited ductile behaviour and showed the lowest pre- and post-slope values compared to stiffer mixtures (plastomers, compound of microfibres and polymers). However, the force required to bring the sample to failure in the first subset of mixtures was relatively lower compared to the latter group. Time of loading (displacement) and maximum load are equally important while studying the cracking performance at an intermediate temperature. Therefore, the concept of calculating the total work (fracture energy) is essential to explain the combined effect of the rate of failure and the maximum force required at the time of failure. This type of illustration is helpful to provide a more reliable picture of crack initiation potential in real traffic conditions.

The fracture energy was determined to quantify the cracking resistance of AC mixtures. The fracture work method defines fracture energy as the area under load–displacement curves until the specimen is fractured [92,93]. This work will equate to fracture energy if all the work is utilised by crack development and propagation. This technique estimates fracture energy, or more precisely, apparent fracture energy. The calculation of the apparent fracture energy assumes that all the work is being utilised by the crack development and propagation, although this is not always the case, as there are few mechanical and heat losses during testing. The mathematical function is shown as follows:

(3) $$G_{fa}=\frac{1}{b\;\left(D-a\right)}\left[\int_{0}^{{µ}_{0}}P_{1}\left(µ\right)dµ+\int_{{µ}_{0}}^{{µ}_{final}}P_{2}\left(µ\right)dµ\right]$$

where *b* = specimen thickness, *D* = diameter of the specimen, and *a* = width of the notch.

*P*_1_(µ) and *P*_2_(µ) = fitting equations before and after the peak, respectively. 

µ_0_ = displacement at the peak.

µ*_final_*= final displacement that can be selected as the displacement at a cut-off load value where the test is considered at an end (taken as 0.3 kN). 

The flexibility index (FI) was also determined to quantify the cracking resistance of AC mixtures. The FI depicts the basic fracture mechanism and is derived using load–displacement curves produced from the I-FIT technique with fracture energy and slope parameters at the post-peak inflection point [43]. Researchers found that FI can effectively capture changes in the materials and volumetric design of AC mixtures [43,90]. 

The FI is determined by dividing the total fracture energy by the slope at the inflection point of the post-peak load–displacement curve.

(4) $$FI=\frac{G_{fa}}{\left|m\right|}\times A$$

where |*m*| = absolute value of the post-peak slope at the inflection point. 

*G_fa_* = fracture energy and represents the area under the load–displacement curve normalised by fractured area. 

Coefficient *A* = unit conversion factor and scaling coefficient (taken as 0.01 as the default).

The rate of deterioration of various asphalt mixtures before and after failure was studied. Secant modulus is the slope of the failure of the material between the starting point of the test to the peak load, whereas the post-peak slope is the slope taken at the inflection point.

### 3.4. FPBB Test

The four point bending beam testing was adopted in this study according to AASHTO T321-07. The asphalt mixture fatigue life (N_f_) is usually described as the number of cycles needed to produce a 50% reduction in the initial stiffness (flexural) modulus (i.e., the stiffness modulus recorded at the 100th cycle) of an asphalt mixture at a particular strain or stress level. Three beams for each mixture at each condition with dimensions of 63.5 ± 5 mm in width, 50 ± 5 mm in depth, and 390 ± 5 mm in length were prepared. The tests were conducted at 25 °C and a frequency of 10 Hz in a strain-controlled mode (300 and 400 micro strain) and sinusoidal loading. The 50% reduction of the initial flexural modulus was used as a failure criterion. 

#### Data Analysis Technique

The most important aspect while developing the correlation is to choose the most appropriate data analysis technique to represent the actual fatigue properties of individual materials. The ratio of dissipated energy change (RDEC) approach is an energy-based approach. It can remove other types of dissipated energy caused by mechanical work or heat production, making it a useful metric for describing the fatigue phenomenon in asphalt. The RDEC is described as the difference in dissipated energy between two consecutive cycles divided by the first cycle’s dissipated energy:

(5) $$RDEC_{a}=\frac{DE_{a}-DE_{b\;}}{DE_{a}\left(b-a\right)}$$

*RDEC_a_* = the average ratio of changes in dissipated energy between cycles *a* and *b*.

*DE_a_* and *DE_b_* = dissipated energy of cycles *a* and *b*, respectively, which were calculated using Equation (6).

(6) $$W_{i}=\pi \sigma_{i}\varepsilon_{i}\sin\delta_{i}.\;$$

where, *W_i_* = dissipated energy at cycle *i*, *σ_i_* = stress level at cycle *i*, *ε_i_* = strain level at cycle *i*, and *δ_i_* = phase angle at cycle *i*. 

**Figure 4:**
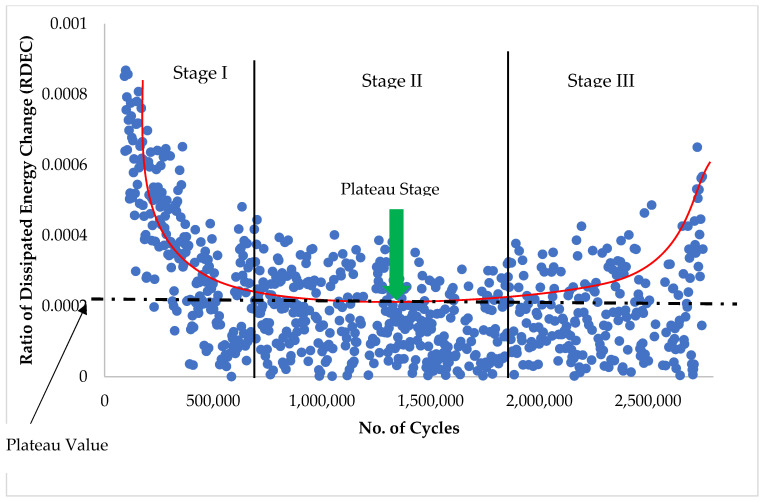
Plastomers RDEC plot using the approach developed by Carpenter et al. [94].

Figure 4 shows the three different phases of a typical RDEC vs. a loading cycle curve from real FPBB test data for the elastomer-modified asphalt mixtures in this study.

**Figure 5:**
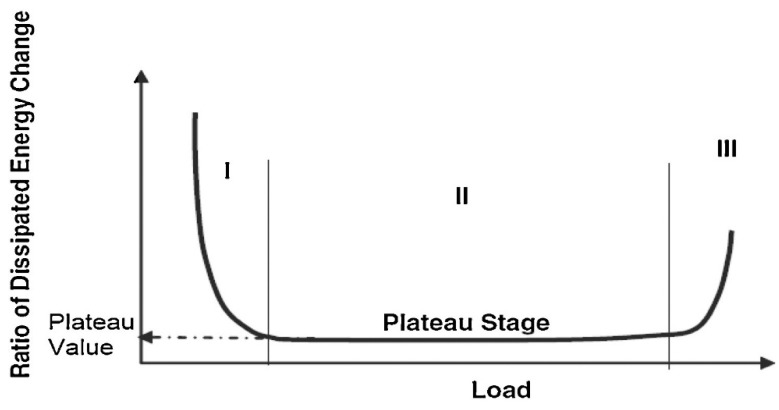
Typical dissipated energy ratio plot with the three characteristic zones [5].

Figure 5 shows three different phases of a typical RDEC vs. a loading cycle curve to simplify the ‘plateau value’ approach. Stage II, wherein the RDEC data remains relatively constant, is of particular importance and is referred to as the plateau value (PV). The plateau stage denotes a phase in which a fixed proportion of input energy is transformed into damage. 

**Figure 6:**
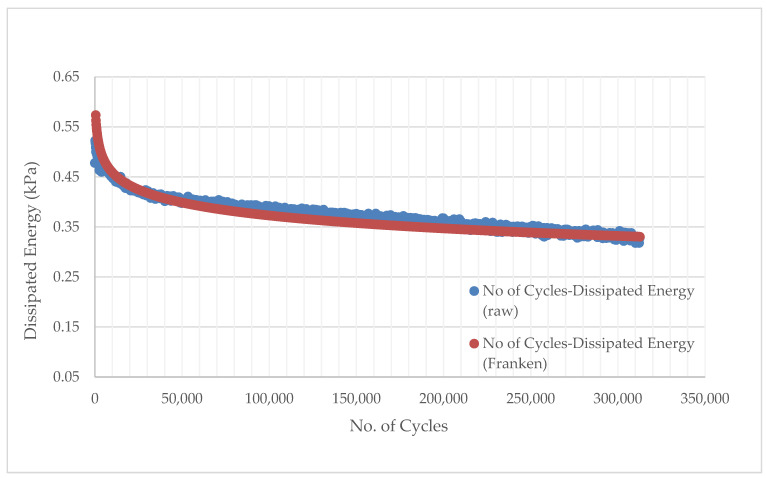
Typical plot between dissipated energy and loading cycles fitted with the Franken model [86].

The advantage of this method over others, such as the cumulative dissipated energy method, is that it eliminates other types of energy that are dissipated during a cyclic fatigue test, such as thermal energy, and focuses only on the dissipated energy damage. The cumulative dissipated energy (CDE) during the plateau stage, where the RDEC value is constant, was used to explain the fatigue life of asphalt mixes in this study. Furthermore, because of the significant variance in the fatigue data, as illustrated in Figure 4, the Franken model [95] was used to fit the No. of cycles versus dissipated energy data to limit noise in the data (Figure 6). 

**Figure 7:**
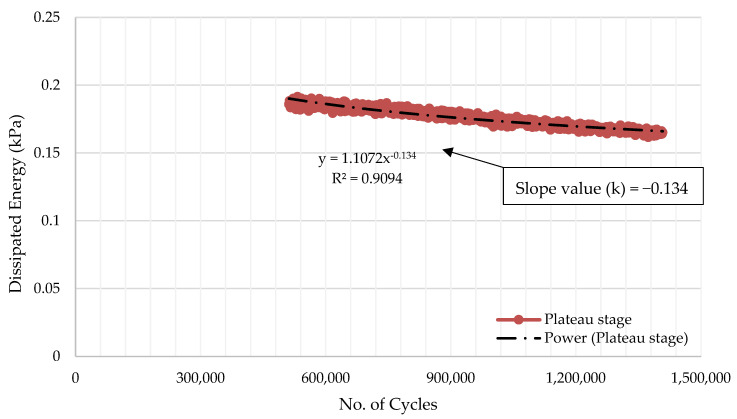
Example of calculation of the exponential slope value (k) of the fitted DE-Loading Cycle for PV computation using Carpenter and Shen’s approach [87].

Carpenter and Shen developed a basic method for performing an RDEC analysis and obtaining the PV from fatigue testing [96]. It entails plotting the dissipated energy (DE) vs. the No. of Cycles (NoC) curve and computing the exponential slope “k” of the DE-NoC curve of the plateau phase using power-law regression to fit the curve (Figure 7). 

Equation (7) is used to compute PV, defined as the RDEC value at the 50% stiffness reduction failure point (*N_f_*_50_).

(7) $$PV=\frac{1-{\left(1+\frac{100}{Nf_{50}}\right)}^{k}}{100}$$

*PV* is a comprehensive damage factor that considers material properties and loading parameters, making it a helpful energy parameter for describing HMA fatigue behaviour [97]. The lower the *PV* in a strain-controlled test, the greater the fatigue life for a particular HMA mixture [97].

### 3.5. Method of Statistical Analysis

#### Linear Regression Analysis

A linear regression analysis was conducted with a 95% confidence level to model the correlation between the bitumen and asphalt mixture fatigue properties. A Shapiro–Wilk normality test and descriptive statistics were used to ensure that the data sets had a uniform distribution before performing a regression analysis. The linearity of the relationships between the different variables was also assessed. An R-Square goodness of fit was also utilised to describe the relationship of the cracking characteristics between binders and the corresponding asphalt mixtures in this research. The backwards elimination technique for choosing dependent variables was used to find the optimum correlating variables and conditions for different combinations of materials [98]. Multiple linear regression analyses were performed by selecting all dependent variables one at a time and then variables with a high *p*-value (i.e., a high *p*-value means a higher probability of the null hypothesis being true if indeed the hypothesis is true) and small t-value (i.e., a small t-value shows the reduced significance of a specific variable). A series of regressions were conducted to remove all dependent variables, except for the one with the lowest *p*-value and highest t-value, which was lastly selected for the final statistical model.

Three replicates for each condition were carried out for binders and asphalt mixtures individually, resulting in a total of six observations for each condition for each type of material. The number of data points in each blend group is given in the statistical analysis of each test segment below. For example, there were six materials in all combinations of materials (All Sources), so the number of observations for each condition was eighteen. 

## 4. Results and Discussion

### 4.1. IDT Test

#### 4.1.1. Statistical Analysis

By grouping all the combinations of unmodified and modified binders—indicated as “All Sources” in Table 7 and grouping all polymer-modified mixes indicated as ”PMB Family” in Table 7—the data show correlation values of 0.78 (LAS: N_f_@5%, IDT: Strength) and 0.93 (LAS: N_f_@2.5%, IDT: Strength), respectively.

This research has confirmed that differences in fracture strength exist for different polymer-modified binders at an intermediate temperature and that fracture strength can be one of the predictors that reasonably correlates with the binders’ fatigue properties. 

Strength properties are highly dependent on the type of binder (i.e., an excessively stiff binder is more prone to contribute to cracking and will fail earlier and vice versa). Therefore, different materials exhibited different strengths based on the material properties at an intermediate temperature and resulted in distinct strength properties. N_f_ by a LAS captured each binder’s distinct fatigue life, which was also linked with the type of polymer modification and grade of bitumen; therefore, the strongest linear correlation exists at an intermediate temperature for the PMB group of materials. The strong correlation suggests that N_f_, which is based on the rheological properties of binders, can help predict the intermediate temperature cracking properties of asphalt mixtures in terms of the indirect tensile strength test. 

**Figure 8:**
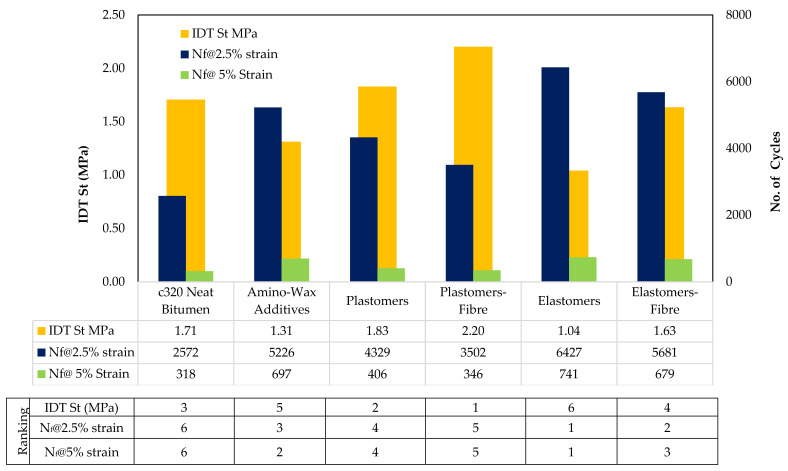
Ranks and Properties of HMA mixes@25 °C for IDT and LAS tests.

The outcomes of the LAS test for lower strain level (2.5%) show less correlation with IDT test results. This can be ascribed to the magnitude of the stresses at 2.5% strain that could not fully activate the complex polymeric structure (simple structural chain folding, strong intermolecular forces, and containing stiffening groups) [99]. As a result, the lower strain levels in the LAS test could not produce a similar damage pattern as in the IDT test, where the loading rate was very high. For the IDT strength, the asphalt mixtures tested were subjected to a high loading rate, which triggered the response from the polymers’ complex structure and better captured the effects of the presence of polymers in the mixes [100] (Fig. 8). However, as the level of strain increased in the LAS test, an improved correlation was observed, probably linked to an accelerated reduction in the fatigue life, as higher strain levels activate the complex structures of the polymers, bringing significant changes in the bituminous matrix. This aspect produced outcomes from the binders’ testing that were more aligned with IDT test results on asphalt mixtures, hence resulting in a good correlation (Figure 8). 

#### 4.1.2. Model Development

A regression analysis was carried out for the most efficient pair of parameters at their best testing conditions to construct the model for predicting the asphalt mixture’s intermediate temperature cracking properties from the binder’s properties. Table 8 presents the results of the regression analysis of different groups of materials.

For instance, to simplify the information given in Table 8, the regression analysis outputs should read as analytical equations as per the examples provided below.

(8) $$IDT\;\left(\mathrm{MPa}\right)=-0.002\;\left(N_{f}@5\%\right)+2.658\;\;\;\;\;\;\;\;\;\;\;\;\;\;\;\;\;\;\;\;\;\;\;\;All\;Sources\;\;$$

(9) $$IDT\;\left(\mathrm{MPa}\right)=2.49\;x\;10^{4}\;\left(N_{f}@2.5\%\right)+3.180\;\;\;\;\;\;\;\;\;\;\;\;\;\;\;PMB\;Family$$

#### 4.1.3. Ranking of binders and Asphalt Mixtures

The binders and mixtures were ranked and were assigned 1 to 6 ranks in Figure 8, where the lowest number represents the best performing and the higher number indicates least performing in cracking. 

### 4.2. SCB Test Results

#### 4.2.1. Statistical Analysis

By grouping, all the combinations of unmodified and modified binders—indicated as “All sources”—the data show the highest correlation value of 0.97 (LAS: N_f@_5%, SCB: Secant Modulus), whereas by grouping all polymer-modified mixes (PMB family), the highest correlation value of 0.98 (LAS: N_f_@5%, SCB: Strength & Secant Modulus) was obtained. Following the ANOVA, a least-square regression analysis was performed to explore trends in the data further. Table 9 shows the relationship between bitumen fatigue parameters and asphalt cracking parameters. 

In general, there was a poor correlation between fracture energy and fatigue life of bitumen for “All Sources” and “PMB Family.” The fracture energy poor correlation can be explained by taking into account the work required before failure of the sample for different materials; for instance, elastomer materials will experience less force at the time of failure than plastomers, which are generally stiffer. On the other hand, the deformation required by elastomers to reach 50% stiffness will be high compared to plastomers, resulting in similar work, as this is the product between force and deformation for all types of materials. Figure 3 is a very good illustration of the fracture energy of all mixtures. Secant modulus and post peak slope are essential considerations while analysing the asphalt cracking data because they give an idea about the material deterioration rate during SCB testing. The slope resulted in the best performing SCB parameter to correlate with the number of cycles of the LAS of bitumen. A material that develops a high slope will ultimately result in faster cracking propagation. As it has been observed, asphalt mixes modified with elastomers sustain a greater number of cycles to failure, as they possess more elastic behaviour under repetitive loading; a similar trend was observed for the post-peak slope. Each material has shown distinct behaviour even after reaching the maximum load, as the load-carrying capacity was transferred to the bitumen and aggregate interface and cohesive properties of bitumen. Materials with more elastic characteristics possess more tensile properties and generate smaller slope values and vice versa. The second-best correlation parameter between LAS and SCB was strength. Plastomers showed higher tensile strength, whereas the elastomer-modified asphalt samples collapsed at lower force values. The strength was negatively correlated with the fatigue life of binders under the constant deformation test (Table 10). 

The flexibility index was also found to be a valuable parameter to predict the fatigue life of bitumen. As FI relies on fracture energy and post-peak slope, materials with high fracture energy and lesser post-peak slope are considered a good performing material. Since the fracture energy value was less variable between various asphalt mixes, the post-peak slope mainly affected the FI value. Asphalt mixes with higher FI performed well in the LAS and resulted in a greater number of cycles and vice versa. Hence, those trends resulted in a good correlation. 

The effect of the test strain level was found similar to what was already discussed in the previous section for the IDT test. A slightly improved correlation is attributed to high strain levels, which mobilise the polymeric chain and lead to permanent changes in the bituminous matrix that are more similar to what is experienced by the asphalt during SCB tests.

#### 4.2.2. Model Development

A regression analysis was carried out for the most efficient pair of parameters at their best testing conditions to construct the model for predicting the asphalt mixture’s intermediate temperature cracking properties from the binder’s properties. Table 10 presents the results of the regression analysis of different groups of materials.

For instance, to simplify the information given in Table 10, the regression analysis outputs should read as analytical equations as per the examples provided below.

(10) $$Secant\;Modulus\left(\frac{\mathrm{kN}}{\mathrm{mm}}\right)=0.013\;\left(N_{f}@5\%\right)+11.223\;\;\;\;\;\;\;\;\;All\;Sources.\;$$

(11) $$Strength\;\left(\mathrm{MPa}\right)=0.001\;\left(N_{f}@5\%\right)+0.858\;\;\;\;\;\;\;\;\;\;\;\;\;\;\;\;\;\;\;PMB\;Family\;\;\;\;$$

#### 4.2.3. Ranking of Binders and Asphalt Mixtures

**Figure 9:**
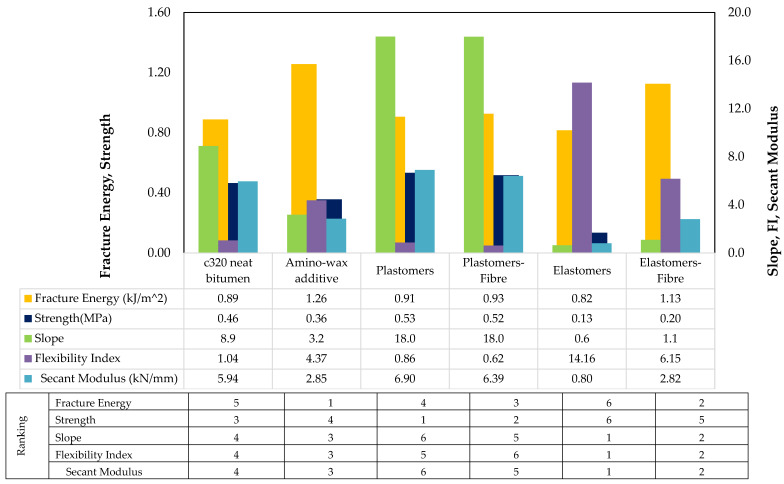
Ranking and properties of HMA mixes@25 °C for the SCB test.

The mixtures were ranked and were assigned 1 to 6 ranks in Figure 9, where the lowest number represents the best performing and the higher number indicates least performing in cracking. Binder ranks based on LAS have already been referred to in Figure 8.

### 4.3. Four Point Bending Beam Test

#### 4.3.1. Statistical Analysis

A statistical analysis (ANOVA) was conducted to identify the bitumen parameters that significantly affected the fatigue cracking potential of asphalt mixtures. A value of “probability > F” less than 0.0500 was used to indicate the existence of a significant effect. N_f_@2.5% and N_f_@5% were found to affect fatigue cracking parameters of asphalt mixtures significantly. N_f_@2.5% of LAS of “all sources” had a greater significant effect on the fatigue cracking potential with a “probability > F” value being less than 0.01. Table 11 shows the relationship between bitumen fatigue parameters and asphalt fatigue parameters.

An improved correlation between LAS testing parameters and FPBBT fatigue indicators was observed compared to LAS parameters and fracture energy in SCB tests. This is probably linked to the different types of dissipated energy linked to the type of testing. In LAS and FPBB tests, the repetition of several loading cycles converts part of the dissipated mechanical energy into heat (thermal energy) due to the viscoelasticity of the material that provides a damping effect and reduces the damage due to fatigue. Cyclic tests on asphalt samples also incorporate a minimal portion of healing due to the self-recovery ability of the asphalt binders that is not considered by other failure tests, such as the SCB, which are mostly focused on strain-energy release and fracture properties. In general, PV showed a better correlation with the LAS ‘N_f_’ parameters than CDE. PV, calculated through the RDEC concept, only considers the portion of the energy that produces crack extension, e.g., without considering the thermal energy or plastic deformation. Similarly, the VECD approach uses continuum damage mechanics based on the work potential to quantify microcracking. Although VECD does not consider the change of time dependency in terms of phase angle, the model is considered to be more similar to the dissipated energy in FPBBT. 

#### 4.3.2. LAS vs. PV

By grouping all the combinations of unmodified and modified binders—indicated as “All Sources”—the data show the highest correlation value of 0.69 (LAS: N_f_@2.5%, FPBBT: PV Value-300) and by grouping all polymer-modified mixes (”PMB family”), the highest correlation value of 0.90 (LAS: N_f_@2.5%, FPBBT: PV Value-400) was obtained. PV value was found to exhibit a good correlation for ”PMB Family” at 400 micro strains compared to 300 micro strains. This can be explained by the intensity of two different strain levels; as the strain levels increase, materials with similar fatigue characteristics start showing more prominent difference of behaviour, as high strains activate the complex structures of polymer-modified binders and produce permanent changes in the microstructure of binders, and the tendency to recover the fatigue damage decreases. At a lower rate of deformation, fatigue curves are missing clear differentiation of the various phases of fatigue damage. At higher strains, materials are subjected to higher force amplitude and exhibit three separate phases of degradation; therefore, the PV value can be captured with more accuracy at high strain, which is why they correlated well at 400 μstrain. Overall, the correlation has been found moderate for “All Sources”, and this could be explained as the binders in various asphalt groups possessed similar properties, resulting in PV values with less variance. However, it should be noted that the PV value is affected by both the slope and the number of cycles. Different materials could possess a similar slope of failure but require different cycles to reach the 50% stiffness reduction. As a result, the PV value can lead to complex behaviours for a similar group of binders with smaller variations involved.

#### 4.3.3. LAS vs. CDE

By grouping all the combinations of unmodified and modified binders—indicated as ”All Sources“—the data show the highest correlation value of 0.51 (LAS: N_f_@2.5%, FPBBT: CDE-300), and by grouping all polymer-modified mixes (PMB family), the highest correlation value of 0.69 (LAS: N_f_@2.5%@, FPBBT: CDE-400) was obtained. In general, there was a poor correlation between CDE-400 and the fatigue life of bitumen for “All Sources” of binders because the CDE value of neat bitumen was quite indifferent due to having a high vulnerability of cracking than the polymer-modified binders. This can be explained by considering the very high dissipated energy produced at 400 μstrain for unmodified bitumen, which indicates the tendency to rebound is very low, and unmodified bitumen is vulnerable to slightly increased traffic loading. In order to resist fatigue cracking, an asphalt binder should be elastic in nature and should dissipate less energy. A high correlation value was obtained for PMB at 400 micro strain because many softer mixes (elastomers and amino-wax-based additives) produced less dissipate energy at 300 μstrain on the beams; however, more cycles resulted from good recovery properties reaching the 50% stiffness reduction.

On the other hand, stiff mixes experienced the tertiary stage of degradation, which indicates they were degrading faster in terms of fatigue properties, so fewer cycles were reported to reach a 50% stiffness reduction. Therefore, resistance against fatigue of different materials at 400 μstrain is quite different, and so the CDE at 400 μstrain was found indifferent, and, ultimately, a comparatively better correlation than 400 μstrain was found. The trend was indifferent at 300 micro strain, where a high correlation value was obtained for all sources and less for PMB sources. This indicates that a lesser strain can help to predict the overall asphalt mixture’s fatigue behaviour using LAS data at 2.5%; however, the complex structure of polymer compounds in the PMB family does not activate at a lesser strain and does not truly characterise the asphalt mixtures.

#### 4.3.4. LAS vs. NoC

By grouping all the combinations of unmodified and modified binders together—indicated as “All Sources”—the data show the highest correlation value of 0.82 (LAS: N_f_@2.5%, FPBBT: No of Cycle-400), and by grouping all polymer-modified mixes (PMB family), the highest correlation value of 0.68 (LAS: N_f_@2.5%, FPBBT: No of Cycle-400) was obtained. The fatigue life of binders and asphalt mixtures was thus correlated with the number of cycles to address the above issue. The correlation was significant. This study counted only those loading cycles to determine the fatigue life of asphalt mixtures involved in the secondary zone (plateau stage). Using the Franken model, the data were fitted. The tangent was drawn at the primary zone (mechanical loss), and another tangent was drawn for the secondary zone. The number of cycles was calculated by deducting the primary zone cycles from the total number at the end of the secondary zone. This technique captured the true fatigue properties of each asphalt mixtures’ type and resulted in a different fatigue life similar to binders determined in the LAS test. Therefore, the number of cycles of the FPBB test in the secondary zone positively correlated with the fatigue life of the LAS test of various binders. Overall, the same trend was observed; as in the case of the PV value and CDE parameter, the high correlation of LAS at 2.5% was observed at 400 micro strain, except “All Sources ”showed the poor correlation of CDE at 400 micro strain.

**Figure 10:**
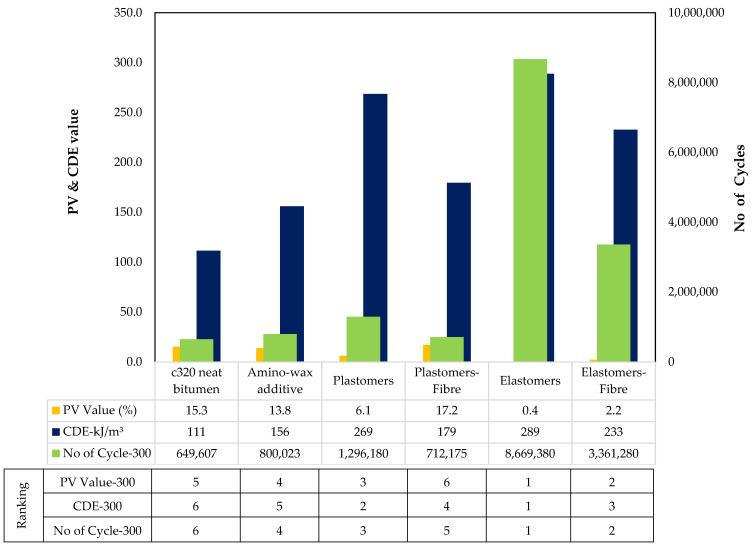
Ranking and properties of HMA mixes@300 μstrain for FBBT tests.

**Figure 11:**
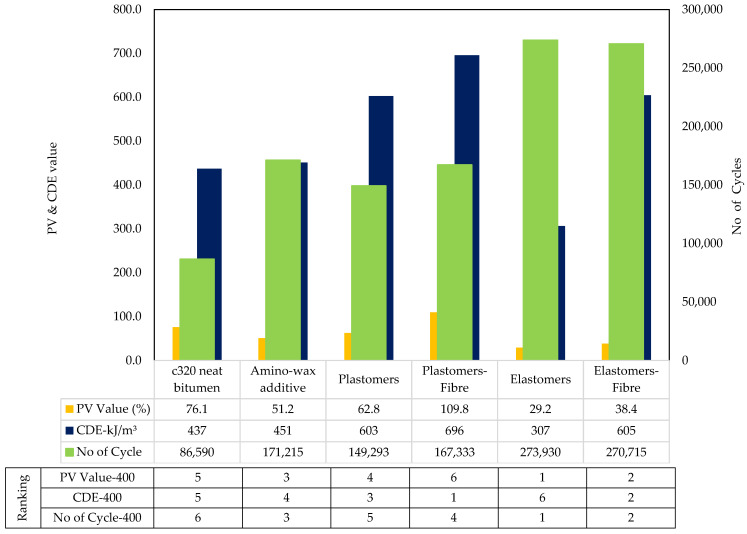
Ranking and properties of HMA mixes@400 μstrain for FBBT tests.

The behaviour of various materials under the FPBB test (repeated loading at a given frequency) is different from the IDT and SCB tests (constant loading rate test) due to differences in the dynamics of the testing methods. The FPBB test is more similar to LAS in the sense that it involves repeated loading cycles at a certain frequency. The correlation of NoC from the FPBB test at 300 and 400 micro strain was moderate to high with the LAS No. of cycles to failure, particularly at 2.5% strain. Furthermore, as it can be inferred from the NoC results (Fig. 10 and 11) of the various asphalt mixtures at 300 and 400 micro strain, the imposed level of strain produced a number of cycles to failure in the order of 10^5^–10^6^, hence suggesting greater strain levels would have been needed to correlate better with LAS tests at 5% strain. The rise from 2.5% to 5% represents a double increase in the strain level for the LAS test on bitumen, whereas shifting from 300 to 400 micro strain for the strain-controlled FPBB test on the asphalt mixture corresponds to a 33% increase. Testing at higher strain levels, particularly for polymer-modified mixtures, is therefore recommended to possibly observe a greater correlation with LAS tests at 5% strain.

#### 4.3.5. Model Development

Regression analyses were carried out for the most efficient pair of fatigue parameters from LAS and FPBB tests at their best testing conditions to construct the model for predicting asphalt mixtures fatigue cracking properties from the binder’s properties. Table 12 presents the results of the regression analysis of different groups of materials.

For instance, to simplify Table 12, the regression analysis outputs should read as analytical equations as per the examples provided below.

(12) $$\mathrm{No}\;\mathrm{of}\;\mathrm{Cycles}@400\;\mu \mathrm{strain}\;=46.17\left(N_{f}@2.5\%\;strain\right)-26,930\;\;\;\;\;\;\;\;\;\;\;All\;Sources$$

(13) $$\mathrm{No}\;\mathrm{of}\;\mathrm{Cycles}@400\;\mu \mathrm{strain}\;=-2.61\;\times \;10^{-4}\;\left(N_{f}@2.5\%\;strain\right)+1.89\;\;PMB\;Family$$

#### 4.3.6. Ranking of Binders and Asphalt Mixtures

The mixtures were ranked and were assigned 1 to 6 ranks in Figure 10 and Figure 11, where the lowest number represents the best performing and the higher number indicates least performing in cracking. Binder ranks based on LAS have already been referred to in Figure 8.

### 4.4. Fatigue Behaviors of Various Materials

For the given binders and asphalt mixtures, fatigue properties can be explained as follows:

PV value is an important consideration while analysing the fatigue data because it helps to give an idea about the material deterioration rate during the fatigue cracking phenomenon. PV value relies on the slope of the secondary zone and the total number of cycles required to reach a 50% stiffness reduction. Having a high PV value is an indication of rapid deterioration of the material and vice versa. Materials with a high potential of propagating the cracking were found to have a high PV value because of the high rate of deterioration during fatigue testing, resulting in fewer cycles to reach a 50% stiffness reduction.

#### 4.4.1. N_f_ and PV Value

Mixed Modified with Elastomers: For mixtures with elastomers and elastomer/fibre, the fatigue life increased by 1234% and 417% at 300 μstrain and 216% and 212% at 400 μstrain, respectively. Hence, the effect of elastomer modification was found beneficial in FPBB tests. Similarly, the PV value for elastomer- modified materials was reduced, which supports a lower deterioration rate (longer fatigue life) under repetitive loading, making elastomers more favourable to resist fatigue.

Mixed Modified with Plastomers: For mixtures with the plastomers and plastomers/fibre, the fatigue life increased by 99% and 10% at 300 μstrain and 72% and 100% at 400 μstrain. Similar trends were found for the PV value of plastomer-modified materials. The PV value for plastomer-modified materials increased, which suggests a greater deterioration rate (lesser fatigue life) under repetitive loading, making plastomers less favourable to cope with fatigue than elastomers. However, the literature already mentioned how fewer cycles to fatigue are reported under constant strain mode for plastomer-modified asphalt. Therefore, the cumulative dissipated energy approach was investigated to further study the fatigue performance of this material.

#### 4.4.2. CDE Value

In order to resist fatigue cracking, an asphalt binder should be elastic (able to dissipate energy by rebounding and not cracking) but not too stiff (excessively stiff materials crack rather than deform then rebound). Therefore, the dissipated energy per cycle should be minimal to resist fatigue.

Mixed Modified with Elastomers: For mixtures incorporating elastomers and elastomer/fibre, the CDE value decreased by −80% and −59% at 300 μstrain and −78% and −56% at 400 μstrain, respectively. Hence, the effect of elastomer modification positively affected FPBB fatigue properties. 

Mixed Modified with Plastomers: For mixtures with plastomers and plastomers/fibre, the CDE value was 21% and 47% at 300 μstrain and −20%, −17% at 400 μstrain, respectively.

### 4.5. Relationship between Cracking Tests

The comparison between rankings of monotonic cracking tests (IDT and SCB) and dynamic tests (FPBB test) was made to evaluate the cracking potential of various asphalt mixtures at an intermediate temperature. Rankings given by IDT strength and SCB fracture strength parameters were mostly similar. The slight difference can be linked to the shape of the sample, the effect of the notch, and the rate of loading. Recent studies have suggested that the SCB test involves a fracture-based mechanistic approach that is considered more advanced to determine the cracking potential compared to IDT testing. SCB and FPBB tests were compared to see the effectiveness of cracking characterisation, despite these testing techniques being different.

Various performance indicators calculated based on the SCB test results, i.e., fracture energy (Gf), flexibility index (FI), fracture strength, and slope and secant modulus were compared with the FPBB test results. Based on the FPBBT results, it was shown that the ranking between the six asphalt mixtures considered in this study correspond to those given by the SCB test parameters, except for the indicator ‘fracture energy’. These findings are similar to the recent study by Aksel Seitllari et al. [44], where the fatigue test (uniaxial tension–compression) results were compared with SCB test performance indicators. The results of the research confirmed the capability of the SCB test to discriminate the mixes for their cracking potential.

Fracture energy in SCB tests may provide a different ranking, as it is controlled by two different components: maximum load and deformation. Therefore, a probability exists where two asphalt mixtures modified with different families of polymers can produce similar fracture energy. For example, elastomers, plastomers, plastomers-fibres, and neat bitumen showed almost similar fracture energy, despite a different failure load and deformation at failure. For other SCB parameters, the difference within polymeric families and types of asphalt mixtures is significant, making them a reliable indicator to differentiate between mixes.

Fracture energy is also affected by the sample geometry, especially in the case of heterogeneous material, such as asphalt concrete [101].

## 5. Summary of Results

Several binders (unmodified and modified with different polymers/additives) and their corresponding asphalt mixes were studied in this research. The testing matrix was explicitly designed to assess the binder’s fatigue performance at an average pavement temperature through a rheological experiment (linear amplitudes sweep test) of binders and determination of load-induced cracking potential (crack initiation and propagation) of the asphalt mixtures through indirect tensile strength, semi-circular bending, and four point bending beam tests. The goal was to compare and assess the relationship between binders and related asphalt mixture properties using various laboratory techniques and materials. Conventional cracking parameters of IDT and SCB, namely tensile strengths, fracture strength, fracture energy, and newer ones such as PV, CDE, and N_f_ of FPBBT, explicitly designed to capture true fatigue behaviour were compared. Secant Modulus through SCB emerged as one of the best potential indicators to enhance the correlation of all tested binders and asphalt mixes. Overall, fatigue parameters through FPBBT have proven the best equipped for predicting asphalt fatigue performance because they can simulate the true fatigue phenomenon of the field in the laboratory, i.e., cyclic loading with constant stress/strain modes to simulate real traffic conditions than the conventional SCB and IDT tests. However, some test conditions’ sensitivity (e.g., method of testing and levels of applied strain) need to be investigated further to achieve better correlations with binders—particularly for plastomer-modified binders.

The strain levels of the rheological experiments were found not to significantly impact the correlation with the asphalt crack initiation testing’s parameters; both 2.5% strain and 5% strain were observed to provide more significant correlation coefficients. However, the correlation coefficients were impacted with high strain levels of LAS when correlating with fatigue cracking properties. The most remarkable correlation of the “All Sources binders” was found with the SCB test. For instance, the N_f_ at 5% of LAS correlated better with the secant modulus than IDT and FPBBT fatigue parameters at 25 °C. Interestingly, the fracture energy of SCB identified the elastomer modified (SBS) blends as the worst-performing material within the tested polymers. In reality, the SCB test, because of the high constant strain mode of testing, could not capture the bitumen recovery component that occurs during the true fatigue phenomenon in the field, making the SCB test not compatible with the fatigue properties of the field.

PV value and loading cycles of the plateau stage of the FPBB test exhibited a moderate to strong correlation with the binder’s fatigue life for both “All Sources” and “PMB family” (R^2^ greater than 0.60). The strongest correlation (R^2^ = 0.82) for N_f_ at 50% of asphalt residual stiffness at 400 μstrain vs. N_f_ at 2.5% of binders was found when grouping all tests conducted on “All Sources” (both elastomer and plastomers), probably due to the heterogeneity of the materials tested, which both LAS and FPBBT captured. On the other hand, the CDE of “All Sources” and “PMB family” was weakly correlated (i.e., R^2^ was almost zero) with the binder’s fatigue life (R^2^ = 0.21–0.69). A lower correlation of “All Sources” at 400 μstrain was because of the high constant strain mode of testing (high stresses were involved), which expedited the rate of deterioration and produced permanent changes in the asphalt mixtures (unmodified and excessive stiff mixtures); this reduced the recovery properties resulting in premature failure (fewer cycles). On the other hand, the fracture energy of the FPBB test at 300 μstrain was not found as a representative for the “PMB family”, as the fracture energy was the product of the stresses and number of cycles, which was underestimated for soft materials. Similarly, the bitumen–asphalt test’s statistical correlation seemed weak between LAS fatigue life and No. of cycles at 300 μstrain and PV value at 300 μstrain of asphalt mixtures (All Sources and PMB Family).

Although the use of No. of cycles, PV value, and CDE in FPBBT ranked the SBS-modified binders as the top-performing material, the SCB and IDT tests assigned SBS-modified asphalt a very low ranking, hence favouring stiffer materials when it comes to testing the asphalt material under very high constant strain. However, the ranking based on FPBBT is closer to those based on LAS fatigue life than IDT and SCB parameters.

The available data indicate that the fatigue life of binders correlates with the fatigue life for mixes made from different polymers and unmodified binders but have similar air voids, sources of aggregates, and gradation. It is possible to design a mixture with a fatigue life that endures a set number of repetitions with the aid of a correlation, as shown in the above table. The amount of data obtained in this investigation was insufficient to permit broad conclusions; however, the results indicate that fatigue life favours the elastomers and underestimates the plastomers’ potential for constant strain fatigue tests. However, it is felt that reliable correlations that will indicate fatigue susceptibility can be developed for asphaltic mixtures.

It should be recognised that future studies should pay attention to the following issues to enhance the correlation between binders and asphalt mixture fatigue testing: (1) the difference between a constant strain and constant stress mode tests for FPBB; (2) the use of the lesser rate of deformations for SCB and IDT as a further expression of asphalt cracking resistance; (3) the use of a variety of bitumen content, air voids, and aggregate gradations; and (4) testing at various ageing conditions for bitumen and asphalt mixtures to consider the short- and long-term ageing.

## Figures and Tables

**Table 1:** Correlations between the properties of bitumen from conventional tests and cracking properties of asphalt mixtures from various tests.

Binder Test/Parameter	Asphalt Mixtures test/Parameter	R^2^	Interpolation	Material Type
Binder Percentage versus Asphalt Mixtures Tests				
Binder content (%)	ε^6^ (strain level required for 1 mil cycles fatigue life in FPBB test) [Testing conditions: 20 °C & 10 Hz]	0.58	[71]	Conventional Warm Mix Asphalt Modifiers: (Advera, Evotherm, and Sasobit: 4.8%, 0.5%, and 1.5% by mass of binder, respectively)
Binder content (%)	N_100_ (Number of cycles (NoC) at a strain level of 100 × 10^−6^ in FPBB test) [Testing conditions: 20 °C & 10 Hz]	0.16		
Asphaltenes (%) from SARA analysis	Material Fatigue Sensitivity (MFS) from S-VECD test [Testing conditions: 18 °C & 10 Hz]	0.50	[72]	Conventional RAP ^1^ (20% & 40% RBR ^2^) RAS ^3^ (20% RBR)
	Critical strain energy release rate (J_c_) from SCB test [Testing conditions: 25 °C]	0.38		

RAP ^1^—Reclaimed Asphalt Pavement, RBR ^2^—Reclaimed Binder Ratio—the percentage of RAP binder by weight with respect to the total binder by weight in the asphalt mix, RAS ^3^—Reclaimed Asphalt Shingles.

**Table 2:** Correlations between the properties of the bitumen from various miscellaneous tests and cracking properties of asphalt mixtures from various tests.

Binder Test/Parameter	Asphalt Mixtures Test/Parameter	R^2^	Interpolation	Material Type
Binder Miscellaneous Properties vs. Asphalt Mixtures Tests				
Displacement at max loading from SENB * [Testing conditions: 5 °C]	Displacement at max loading from Fénix test [Testing conditions: 5 °C]	0.92	[73]	Two neat (PG 64-22 & PG 76-22) Two modified binders (PG 76-22 & PG 76-28).
RTFO ** aged-percent recovery(%R) [ASTM D6084 ) Method A]	CTOD **** (mm) from DENT test [Testing conditions: 25 °C]	0.00	[74]	PG64-28 (Control) PG64-28+PPA PG64-34+SBS PG76-22+SBS PG64-22+12% GTR PG64-28+Latex (2%)
%R from MSCR *** Test [Testing conditions: high PG temperature of binder & stress of 100 Pa]		0.41		
R % from MSCR Test [Testing conditions: high PG temperature of binder & stress of 3200 Pa]		0.77		
Critical temperature difference (ΔTc (°C)) from the BBR test	J_c_ from SCB test [Testing conditions: 25 °C]	0.77	[72]	Conventional RAP (20% & 40% RBR) RAS (20% RBR)
	MFS from S-VECD test [Testing conditions: 18 °C & 10 Hz]	0.57		

SENB *—single-edge notched beam, RTFO **—Rolling thin film oven, MSCR ***—Multiple stress creep recovery, CTOD ****—critical tip opening displacement.

**Table 3:** Correlations between the properties of the bitumen from time sweep tests and cracking properties of asphalt mixtures from various tests.

Binder Test/Parameter	Asphalt Mixtures Test/Parameter	R^2^	Interpolation	Material Type
Time Sweep vs. Asphalt Mixtures Tests				
Cycles to failure (Np) [Testing conditions: 30 °C & 15 Hz]	Dissipated energy (N·m) to failure from IDT [Testing conditions: 30 °C]	0.94	[15]	Conventional (PG * 58-22) Crumb rubber (CR) modified asphalt mixtures (CR at 3%, 6%, 9%, 12%, and 15% by mass of binder)
	Indirect Tensile Strength (MPa) from IDT [Testing conditions: 30 °C]	0.45		
NoC at 50% reduction in G* [Testing conditions: 24–32 °C & 30 Hz to 0.01 Hz]	NoC at 50% reduction in G* from FPBB test [Testing conditions: 24–32 °C & 10 Hz]	0.66–0.90	[27]	Conventional Elastomer-modified Oxidised binder (PG 82-22) Plastomer-modified (PG 82-22 & 76-22)
Fatigue life (N_f_) [Testing conditions: 20 °C & 1,5,10 Hz]	N_f_ from FPBB test [Testing conditions: 20 °C & 10 Hz]	0.98	[75]	Conventional plant-produced mixture Plant-produced mixture with 35% RAP
N_p20_ ** at 5.0% strain [Testing conditions: 12.1 °C 8.6 °C 6.2 °C & 10 Hz]	N_f_ from FPBB test [Testing conditions: 12.1 °C 8.6 °C 6.2 °C & 10 Hz ]	0.31–0.54	[76]	PG 64-28 (Styrene-Butadiene Styrene Rubber) PG 58-34 (Ethylene Ter-polymer) PG 64-34 (Ethylene Ter-polymer)
Stiffness Modulus (G*)	N_f_ from FPBB test (AASHTO T321)	0.04	[77]	Mixtures with 25% and 50% RAP Mixtures with 25% and 50% RAP along with rejuvenator (7.5% by mass of the recycled asphalt binder)

PG *—Performance Grade, N_p20_ **—NoC when the dissipated energy ratio detracts 20 percent from the equality line.

**Table 4:** Correlations between the properties of the bitumen from frequency sweep tests and cracking properties of asphalt mixtures from various tests.

Binder Test/Parameter	Asphalt Mixtures Test/Parameter	R^2^	Interpolation	Material Type
G*·Sinδ * from Frequency Sweep Test vs. Asphalt Mixtures Tests				
G*·Sinδ [Testing conditions: 30 °C]	Dissipated Energy (N·m) to failure from IDT [Testing conditions: 30 °C]	0.68	[15]	Conventional (PG 58-22) Crumb rubber (CR) modified asphalt mixtures (CR at 3%, 6%, 9%, 12%, and 15% by mass of binder)
	Indirect Tensile Test Strength (MPa) from IDT [Testing conditions: 30 °C]	0.90		
G* Sinδ [Testing conditions: 24–32 °C & 15 °C]	N_f_ from FPBB test [Testing conditions: 24–32 °C & 10 Hz]	0.23	[27]	Conventional Elastomer-modified Oxidised binder (PG 82-22) Plastomer-modified (PG 82-22 & 76-22)
G* Sinδ [Testing conditions: 25 °C]	N_f_ from FPBB test [Testing conditions: 25 °C & 10 Hz]	0.60	[78]	PG 58-22 and PG 64-22 Mixtures of PG 58-22 and PG 64-22 modified with 4%, 8%, and 12% gilsonite, and 3% and 5% SBS (by mass of binder)
	RDEC ** from FPBB test [Testing conditions: 25 °C & 10 Hz]	0.59		

G*·Sinδ *—SHRP fatigue parameter, RDEC **—Ratio of dissipated energy change.

**Table 5:** Correlations between the properties of the bitumen from LAS tests and cracking properties of asphalt mixtures from various tests.

Binder test/Parameter	Asphalt Mixtures Test/Parameter	R^2^	Interpolation	Material Type
LAS vs. Asphalt Mixtures Tests				
NoC to failure [Testing conditions: intermediate PG temperature]	Ratio between LTPP * Cracked Area & Pavement Thickness [Testing conditions: intermediate PG temperature]	0.64	[38]	Eight LTPP binders
Damage parameter determined using VECD analysis of strain sweep data at 25% reduction in G* [Testing conditions: 12.1 °C 8.6 °C 6.2 °C & 10 Hz ]	N_f_ from FPBB test [Testing conditions: 12.1 °C 8.6 °C 6.2 °C & 10 Hz ]	0.98–0.99	[76]	PG 64-28 (Styrene-Butadiene Styrene Rubber) PG 58-34 (Ethylene Ter-polymer) PG 64-34 (Ethylene Ter-polymer)
A_35_ value (fatigue life of binder expressed as number of cycles) [Testing conditions: 20 °C]	N_f_ from FPBB test [Testing conditions: 20 °C & 10 Hz]	0.85–0.96	[79]	Conventional Mixture modified with 6%LDPE Mixtures with nano clay additives at 3 different percentages
Cumulative dissipated energy [Testing conditions: 20 °C]		0.92–0.95		
Predicted Binder Fatigue life using S-VECD model [Testing conditions: 18 °C]	Field N_f_@25 metres (m) cracking formulation from FHWA **-ALF ***[Testing conditions: 18 °C]	0.98	[80]	PG 64-22 PG 64-22 STA PG 64-22 LTA CR-TB Terpolymer SBS-LG
Binder Fatigue life coupled with S-VECD analysis [Testing conditions: 19 °C]	Fatigue life from controlled crosshead (CX) cyclic direct tension tests [Testing conditions: 19 °C]	0.84	[81]	PG 70-22 Mixtures modified with Evotherm 3G (0.5% by weight of total asphalt) Mixtures modified with foaming additive (0.7% by weight of total asphalt)
LAS—‘A’ parameter [Testing conditions: 19 °C]	Fatigue cracking from LTPP measurements [Testing conditions: 19 °C]	0.93	[82]	PG 64-28 PG 64-28-sbs PG 58-34 Elvaloy PG 64-34 Elvaloy
LAS—‘A’ parameter [Testing conditions: intermediate PG temperature]	CTOD **** (mm) from DENT test [Testing conditions: 25 °C]	0.00	[74]	PG64-28 (Control) PG64-28+PPA PG64-34+SBS PG76-22+SBS PG64-22+12% GTR PG64-28+Latex (2%)
LAS—‘B’ parameter [Testing conditions: intermediate PG temperature]		0.32		
N_f@_2.5% strain [Testing conditions: intermediate PG temperature]		0.48		
N_f_@5% strain [Testing conditions: intermediate PG temperature]		0.66		
N_f@_ 3, 4, 5% strain [Testing conditions: 25 °C]	N_f_ from FPBB test [Testing conditions: 25 °C & 10 Hz]	0.94	[78]	PG 58-22 and PG 64-22 Mixtures of PG 58-22 and PG 64-22 modified with 4%, 8%, and 12% gilsonite, and 3% and 5% SBS (by mass of binder)
	RDEC from FPBB test [Testing conditions: 25 °C & 10 Hz]	0.95		
LAS—‘A’ parameter [Testing conditions: 25 °C]	N_f_ from FPBB test [Testing conditions: 25 °C & 10 Hz]	0.86		
LAS—‘A’ parameter [Testing conditions: 25 °C]	RDEC from FPBB test [Testing conditions: 25 °C & 10 Hz]	0.90		
N_f_@2.5, 3.5 and 4% strain	N_f_ from FPBB test@500, 700, 800 μstrain	0.87	[77]	Mixtures with 25% and 50% RAP Mixtures with 25% and 50% RAP along with rejuvenator (7.5% by mass of the recycled asphalt binder)
N_f_@2.5% strain	IDT fatigue testing (ITFT)	0.93		
N_f_ [Testing conditions: 25 °C]	N_f_ from FPBB test [Testing conditions: 25 °C]	0.99	[75]	Conventional plant-produced mixture Plant-produced mixture with 35% RAP
		0.95		
N_f_ [Testing conditions: 20 °C]	N_f_ from FPBB test [Testing conditions: 20 °C]	0.99	[83]	VG 10 and VG 30 Mixtures modified with EVA and SBS
N_f_ [Testing conditions: 20 °C]	N_f_ from FPBB test [Testing conditions: 20 °C]	0.99	[6]	Mixtures with neat 30/45 binder Mixtures with 3.0% and 7.5% SBS modified binder
N_f_ [Testing conditions: 20 °C]	N_f_ fromITFT test [Testing conditions: 20 °C]	0.37–0.98	[84]	Control Mixtures modified with 3%, 6%, and 9% Siliceous additives Mixtures modified with 3%, 6%, and 9% date seed ash (DSA) Mixtures modified with 3%, 6%, and 9% limestone
A_35_ [Testing conditions: 20 °C]	N_f_ from FPBB test [Testing conditions: 20 °C]	0.68	[10]	Conventional RAP (20% & 40% RBR) RAS (20% RBR) RAS & RAP mixtures modified with Evotherm and water foam technologies
A_35_ [Testing conditions: 20 °C]	N_f_ from FPBB test [Testing conditions: 20 °C]	0.863	[85]	AC-60/70 Mixtures modified with 2, 4, 6, and 8 percent of nano silica

LTPP *—long-term pavement performance, FHWA **—Federal Highway Administration, ALF ***—Accelerated Loading Facility, CTOD ****—critical tip opening displacement.

**Table 6:** Types of material.

Name of Polymers/Additives	Nomenclature Used in This Study	Type of Polymers/Additives [4]	Percentage of Polymers/Additives	Method of Blending [4]
Amino-Wax Based Additives	-	Amino derivatives in liquid form; Density (25 °C) = 0.95–1.05 g/cm^3^; Viscosity (25 °C) = 150–250 cP	0.3% by weight of neat bitumen (C320)	Heating of aggregates (145 °C) and bitumen (140 °C) Addition of additives in bitumen + blending for 20 min using a shear mixer Pouring modified bitumen into aggregates + mixing at 140 °C (5 min) Addition of filler into aggregates + mixing at 140 °C for 10 min
Plastomer-Fibres	Fibres Family	Proprietary blend (pellet form) of cellulose/glass fibres and plastomeric (PE) polymers	0.3% by weight of aggregates	Heating of aggregates (180–185 °C) and bitumen (140 °C) Addition of additives into aggregates in mixer + blending at 175–185 °C (10 min) Pouring C320 bitumen into the aggregates + mixing at 175–185 °C (5 min) Addition of filler into the aggregates + mixing at 175–185 °C (10 min)
Elastomer-Fibre		Proprietary blend (pellet form) of cellulose fibres and elastomeric polymers (SBS.)		
Plastomers	Plastomers	A compound of polyethylene-based plastomers	6% by weight of neat bitumen (C320)	
Commercially available SBS-modified bitumen	Elastomers	Styrene-butadiene-styrene (SBS); 70:30 styrene/butadiene ration (linear)	Industrially modified with approx. 6% of SBS (by weight of binder)	Heating of aggregates (180–185 °C) and elastomer (SBS) modified bitumen (180 °C) Pouring modified bitumen into the aggregates + mixing at 180–[0–9] at 185 °C (5 min) Addition of filler into the aggregates + mixing at 180–185 °C (10 min)

**Table 7:** Pearson coefficients of determinations between binders LAS and asphalt mixtures IDT@25 °C. (Green Background = Moderate to High Correlation (Darker Green = Higher Correlation Compared to Lighter Green); Yellow Backgound = Average Correlation;).

Correlated Data		Bitumen Test Parameters	Relationship Type	Asphalt IDT Test Parameters	
				All Sources ^1^	PMB Family ^2^
Bitumen Test	Asphalt Mixture Test			Sample Size for s!Tatistical Analysis	
				36	30
				R^2^ Value	
LAS	IDT	N_f_@2.5% Strain	linear *y* = *ax* + *b*	0.67	0.88
		N_f_@5% Strain		0.78	0.93

**All sources ^1^**—The group includes plastomers, elastomers, unmodified bitumen, polymeric compounds with microfibres and amino-wax based mixes, **PMB Family ^2^**—The group includes plastomers, elastomers, amino-wax based and polymeric compounds with microfibres.

**Table 8:** Best correlated parameters and testing conditions between a LAS test@25 °C on bitumen and IDT@25 °C on asphalt mixtures.

Mix ID	Bitumen Testing Parameter	Asphalt Testing Parameter	R^2^	Adjusted R^2^	t Stat	*p*-Value	X1 Variable	Intercept
All Sources	N_f_@5%	IDT	0.785	0.742	−4.271	0.008	−0.002	2.658
PMB Family	N_f_@2.5%	IDT	0.927	0.902	−6.152	0.009	0.000249	3.180

**Table 9:** Pearson coefficients of determinations between binders’ LAS and asphalt mixtures’ SCB tests; (Green Background = Moderate to High Correlation (Darker Green = Higher Correlation Compared to Lighter Green); Yellow Backgound = Average Correlation; Red Background = Poor Correlation).

Correlated Data					Bitumen Parameters—LAS test			
Bitumen Test	Asphalt Mixture Test	Temperatures	Relationship Type	Asphalt SCB test Parameters	N_f@_2.5% Strain	N_f_@5% Strain	N_f_@2.5% Strain	N_f_@5% Strain
					Sample Size for Statistical Analysis = 36		Sample size for Statistical Analysis = 30	
					All Material Sources		PMB Family	
					R^2^ Value			
LAS	SCB test	25 °C	linear *y* = *ax* + *b*	Fracture Energy (kJ/m^2^)	0.24	0.4	0.25	0.28
				Strength (MPa)	0.8	0.85	0.92	0.97
				Slope	0.64	0.8	0.95	0.98
				Flexibility Index	0.71	0.67	0.75	0.77
				Secant Modulus (kN/mm):	0.94	0.97	0.98	0.98

**Table 10:** Best correlated parameters and testing conditions of bitumen LAS parameter with SCB@25 °C).

Mix ID	Bitumen Testing Parameter	Asphalt Mixture Testing Parameter	R^2^	Adjusted R^2^	t Stat	*p*-Value	X_1_ Variable	Intercept
All Sources	N_f_@5%	Secant Modulus	0.970	0.963	−12.609	0.000	−0.013	11.223
PMB family	N_f_@5%	Strength	0.974	0.974	−10.620	0.002	−0.001	0.858

**Table 11:** Pearson coefficients of determinations between asphalt binders and mixtures for FPBBT; (Green Background = Moderate to High Correlation (Darker Green = Higher Correlation Compared to Lighter Green); Yellow Backgound = Average Correlation; Red Background = Poor Correlation).

Correlated Data					Bitumen LAS test			
Bitumen Test	Asphalt Mixture Test	Temperature	Relationship Type	Asphalt Mixture FPBB Test Parameters	N_f_@2.5% Strain	N_f_@5% Strain	N_f_@2.5% Strain	N_f_@5% Strain
					All Sources		PMB Family	
					Sample Size for Statistical Analysis 36		Sample Size for Statistical Analysis 30	
					R^2^ Value		R^2^ Value	
LAS	Four Point Bending Beam@300 and 400 micro strain	25 °C	linear *y* = *ax* + *b*	PV Value-300	0.63	0.42	0.60	0.30
				CDE-300	0.51	0.20	0.21	0.02
				No of Cycle-300	0.58	0.43	0.65	0.38
				PV Value-400	0.69	0.66	0.90	0.78
				CDE-400	0.14	0.25	0.69	0.63
				No of Cycle-400	0.82	0.66	0.68	0.53

**Table 12:** Best correlated parameters and testing conditions of bitumen LAS@25 °C with asphalt mixture; Four Point Bending Beam tests at 300 μstrain and 400 μstrain@25 °C.

Mix ID	Bitumen Testing Condition	Asphalt Mixture Testing Parameter	Asphalt Mixture Testing Condition	R^2^	Adjusted R^2^	t Stat	*p*-Value	X_1_ Variable	Intercept
All Sources	N_f_@2.5% strain	No of Cycles@400	25 °C	0.82	0.78	4.28	0.01	46.17	−26,930
PMB Family	N_f_@2.5% strain	PV Value-400	25 °C	0.90	0.87	−5.2	0.01	−2.61 × 10^−4^	1.89

## Data Availability

The data that support the findings of this study are available from the corresponding author upon reasonable request.

## List of Acronyms

LTPP	Long-term pavement performance
MFS	Material fatigue sensitivity
CTOD	Critical tip opening displacement
DE	Dissipated energy
FE	Fracture energy
ALF	Accelerated loading facility
CDE	Cumulative dissipated energy
MSCR	Multiple stress creep recovery
N100	Number of cycles at a strain level of 100 × 10^−6^ in FPBB test
Nf	Number of cycles to failure (LAS test)
NoC	Number of cycles to failure (FPBB test)
PG	Performance grade
PV	Plateau value
RAS	Reclaimed asphalt shingles
RBR	Reclaimed binder ratio
RDEC	Ratio of dissipated energy change
RTFO	Rolling thin film oven
FI	Flexibility index
FPBB	Four point bending beam test
Gfa	Fracture energy
ITS	Indirect Tensile Strength
Jc	Critical strain energy release rate
LAS	Linear amplitude sweep
s.m	Secant modulus
SARA	Saturate, Aromatic, Resin and Asphaltene
SCB	Semi-circular bending
SENB	SENB single-edge notched beam
VECD	Viscoelastic continuum damage
St	Fracture strength
ε^6^	Strain level required for 1 mil cycles fatigue life in FPBB test
